# Patients Leaving Against Medical Advice-A National Survey

**DOI:** 10.5005/jp-journals-10071-23138

**Published:** 2019-03

**Authors:** Gunchan Paul, PL Gautam, Rubina Khullar Mahajan, Nikhil Gautam, Suresh Ragavaiah

**Affiliations:** 1-3,5 Department of Critical Care Medicine, Dayanand Medical College and Hospital, Punjab, India; 4 Department of Psychiatry, Geetanjali Medical College, Rajasthan, India

**Keywords:** Discharge against medical advice, Intensive care unit, Left against medical advice, National survey

## Abstract

**Background:**

Leaving against medical advice (LAMA) is a common health concern seen worldwide. It has variable incidence and reasons depending upon disease, geographical region and type of health care system.

**Materials and methods:**

We approached anesthesiologists and intensivists for their opinion through ISA and ISCCM contact database using Monkey Survey of 22 questions covering geographical area, type of healthcare system, incidence, reasons, type of disease, expected outcome of LAMA patients etc.

**Results:**

We received only 1154 responses. Only 584 answered all questions. Out of 1154, only 313 respondents were from government medical colleges or hospitals while remaining responses were from private and corporate sector. Most hospitals had >100 beds. ICUs were semi-closed and supervised by critical-care physicians. LAMA incidence was maximum from ICU (45%) followed by ward (32%) and emergency (25%). Most patients of LAMA had ICU stay for >1 week (60%). Eighty percent of the respondents opined that financial constraints are the most common reason of LAMA. Unsatisfactory care was rarely considered as a factor for LAMA. Approximately 40% patients had advanced malignancy or disease. Nearly 2/3rd strongly believed that insurance cover may reduce the LAMA rate.

**Conclusion:**

Most patients get LAMA from the ICU after a stay of week. Financial constraints, terminal medical illness, malignancy and sepsis are major causes of LAMA. Remedial methods suggested to decrease the incidence include a good national health policy by the state; improved communication between the patient, caregivers and heathcare team; practice of palliative and end-of-life care support; and lastly, awareness among the people about advance directives.

**How to cite this article:**

Paul G, Gautam PL *et al*. Patients Leaving Against Medical Advice-A National Survey. Indian J Crit Care Med 2019;23(3):143-148.

## INTRODUCTION

The event when a patient already admitted in the hospital wishes to leave against the clinicians' advice is referred to as discharge against medical advice (DAMA) or leave against medical advice (LAMA). The incidence varies from 0.8 to 2.2% of discharges from different medical services at various tertiary care and teaching hospitals across the globe^1,2^. Our experience from a prospective trial conducted at a tertiary care teaching hospital which provides care in a low-middle income country suggested that rates of discharge LAMA were much higher than those reported in the literature^3^. These patients are often acutely ill at the time of self-discharge because of the disease process itself along with incomplete treatment. Thereby increasing the risk of mortality or need for readmission^4,5^. Hence, DAMA poses a major challenge not only for the patients but also for many clinicians who treat these patients.

Leaving against medical advice or discharge against medical advice is a relatively common phenomenon. Scarce data is available in literature on various aspects of the problem like type of cases, reasons and practice patterns of hospitals from where patients leave. The little information available comes from either single centre studies or studies in specific groups of patients especially trauma^1,6^. To get a overview of the problem, it is essential to have data from several institutions to reflect the vast and diverse spectrum of cases that leave the hospital, services provided and practices followed among them. Such information will not only be useful to identify deficiencies in the organization of care but also help to identify targets that may serve to the development of interventions to reduce the rate of patients leaving the hospital prematurely. The data would also provide baseline estimates of the prevalence and severity of the problem. Thus, we planned a multicenter study to gather such information from physicians and intensivists working all over the country.

## MATERIALS AND METHODS

### Study Population

A web-based national survey of Indian anesthesiologists and intensivists was conducted between 1st September 2017 and 15th October, 2017. The survey covered all the domains of hospital infrastructure, working pattern, demographic profile, reasons for going LAMA and expected outcome of these patients.

### Survey Distribution

The survey was distributed via e-mail to anesthesiologists and intensivists for their opinion through ISA (Indian Society of Anaesthesiologists) and ISCCM (Indian Society of Critical Care Medicine) contact database using Monkey Survey on September 4, 2017 and it remained available for 7 days. Three repeat requests were sent weekly via e-mail to non-responders in order to improve the response rate.

### Survey Instrument

The 22-item survey questionnaire (Table 1) was developed by the authors based on the literature review of patients who leave against medical advice. The questionnaire included four sections. The first section (A) captured the demographic information like the geographical area, number of hospital and intensive care unit beds along with average bed occupancy of the hospital and intensive care unit (ICU) of the responding physician. The second section (B) assessed the type of institute (government, private, charitable or corporate) and type of care provided (intensive care consultants/ senior residents/ MBBS residents/ others) by the institute where the respondent worked. The third section (C) assessed the respondents' perceptions with regard to various factors related to LAMA (Table 2):

**Table 1:** List of items of the survey questionnaire along with percentage of responses

*Characteristic*	*Percentage of responses*
*Section A*	
**Type of institute**	
Private hospital	33.28
Corporate Hospital	27.73
Government Hospital	18.20
Private Medical College/institute	9.36
Government Medical College / Institute	6.93
Charitable Hospital	4.51
**Hospital beds**	
Less than 100 beds	24.20
101 - 500	41.18
501 - 1000	20.00
More than 1000	14.62
**ICU Beds**	
Less than 20	41.34
21 - 50	36.13
More than 50	22.52
**Bed occupancy**	
Less than 50%	13.85
50 - 80%	42.57
More than 80%	43.58
**Average length of ICU stay**	
Less than 24 hours	10.79
Less than 1 week	59.93
1 week to 2 weeks	24.14
More than 2 weeks	8.56
*Section B*	
**Type of ICU**	
Closed	14.19
Semi-closed/Semi-open	60.14
Open	25.68
**Type of care provided**	
Critical care consultant available 24 hours in house	40.27
Consultant on call + PG residents/SR round the clock cover	42.30
MBBS Resident with critical care consultant on call	11.00
Other than MBBS doctor with critical care consultant on call	5.25
ICU is primarily covered by other than critical care consultant	6.77
*Section C*	
**Patients leave against medical advice from**	
Emergency room	25.30
Ward	32.82
ICU	62.91
**Gender Bias**	
Predominantly male patients gets LAMA	6.71
Predominantly female patients get LAMA	8.09
No difference	68.85
Cannot comment	16.35
**LAMA patients with health insurance**	
0%	41.57
1 - 10%	49.91
11 -25%	5.39
more than 25%	3.13
**First opinion of LAMA is by**	
Primary physician (to whom the patient first went to)	16.33
Critical care specialist (ICU In-charge)	11.90
Treating Nurse	0.68
Patient's family members	65.82
Cannot answer	5.27
**LAMA patients go to**	
Mostly move to an advanced medical centre	5.93
Mostly move to economical or government hospitals	50.17
Mostly move back to home	9.66
Mostly move back to home and die	22.37
Cannot comment	11.86
*Section D*	
**Role of economic status in LAMA**	
Strongly agree	56.71
Agree	33.96
Disagree	3.90
Strongly disagree	0.85
Do not know	4.58
**Role of iatrogenic complications in LAMA**	
Strongly agree	1.02
Agree	27.33
Disagree	43.46
Strongly disagree	16.13
Do not know	12.05

**Table 2:** Analysis of the reasons of LAMA with survey items of section A

*Reasons*→ *Characteristics* ↓	*Financial constrains*	*Lack of social support*	*Unsatisfied with care*	*Terminal/ critical condition*
Type of hospital	0.00	0.969	0.824	0.769
Hospital beds	0.318	0.424	0.210	0.065
ICU beds	0.085	0.912	0.946	0.607
Type of ICU	0.00	0.001	0.005	0.000
Type of care	0.03	0.040	0.010	0.035

- Hospital area from where maximum patients left the hospital against advice (emergency, ward or ICU)
- Age and gender distribution of the patients going LAMA
- Outcome of the patients in terms of death or discharge
- Percentage of patients covered by medical insurance
- Who initiated discussion of LAMA
- Physician's rating of the outcome of patients going AMA
- Condition of the patient at time of LAMA.

Finally, the fourth section (D) looked into the physician's view point regarding the various probable reasons of LAMA, i.e. economic status, lack of social support, dissatisfaction with care, terminal illness or iatrogenic complications.

The survey was first field-tested by sending it to ten anaesthetists and intensivists within our institute and insights from the tester's responses and oral comments were incorporated into the final survey instrument.

### Analysis

The responses to all questions were presented as percentages. Statistical analysis was done to compare the proportion of patients leaving against medical advice with all the factors proposed among the groups using Statistical Package for the Social Sciences (IBM SPSS Statistics for Windows, Version 20.0. Armonk, NY: IBM Corp.). Statistical significance was defined by *p* value ≤ 0.05.

## Results

**Fig. 1:**
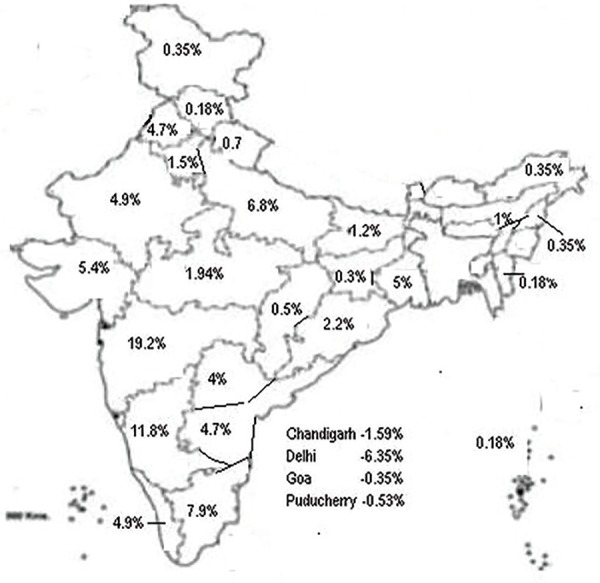
The statewise distribution of responses of the survey (in percentage)

We received 1154 responses out of which only 584 answered all the 22 questions (50.6%). The statewise distribution of the responses is depicted in Figure 1. Maximum responses were from Maharashtra (19.22%) followed by Tamil Nadu (7.94%), Delhi (6.35%) and Gujarat (5.47%). 384 respondents (33.28%) were from private hospitals, 320 (22.73%) from corporate hospitals, 210 (18.28%) from government hospitals, 108 (9.36%) from private medical colleges, 80 (6.93%) from government medical colleges and 52 (4.51%) from charitable hospitals.

Majority (75.8%) of the our data was from hospitals which had >100 beds; 22.52% data being from ICUs with 50-100 beds and 36.13% from ICUs with 20-50 intensive care beds. 60.14% of the respondents worked in ICUs where patient care was provided by both the primary physician and the intensivists. In 40.27% responses, the specialized services were provided by specialists present in the ICU round the clock and in the other 42.30% critical care specialists were on call and ICU was looked after by the post graduate residents/ senior residents. The remaining 22% ICUs were managed by doctors other than critical care specialists.

In this study, 62.91% of patients went LAMA from ICU followed by ward (32.82%) and emergency (25.6%). 82% respondents believed that more than 75% of patients are discharged to ward. The results of our survey with respect to time frame show that only 10% patients went LAMA within 24 hours of admission, 60% of the patients went LAMA within the first week of stay in the hospital/ ICU and remaining 25% during the second week of admission to the hospital. 84.34% respondents believed that LAMA rate from their ICU was within 0-25%. Respondents thought that in our country, most of LAMA initiative was taken by family members (65%) and in 30% cases, they took patient back home and a similar number (30%) to government or charitable hospitals. Respondents also thought that 90% patients who took LAMA did not have health insurance and 70% suggested that reduction in the cost of health services or national health schemes would decrease the incidence of LAMA in this subset of patients.

**Fig. 2:**
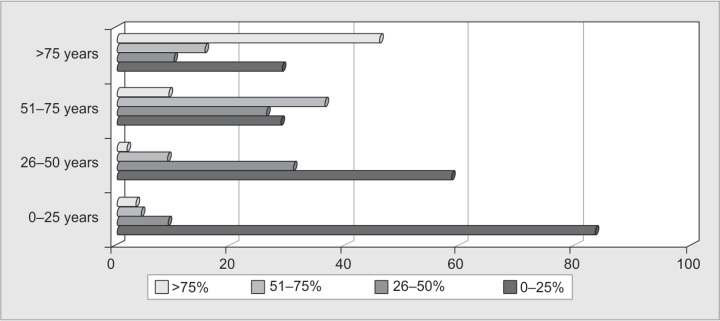
Distribution of LAMA patients of different age categories

In our third section, 83% respondents thought that incidence of LAMA was least in young age (0-25 years) and 46% thought that this incidence increased up to 75% in the elderly population (>75 years) as shown in Figure 2. On comparing sex ratios for LAMA, 70% respondents had the view that there was no gender bias in LAMA.

**Fig. 3:**
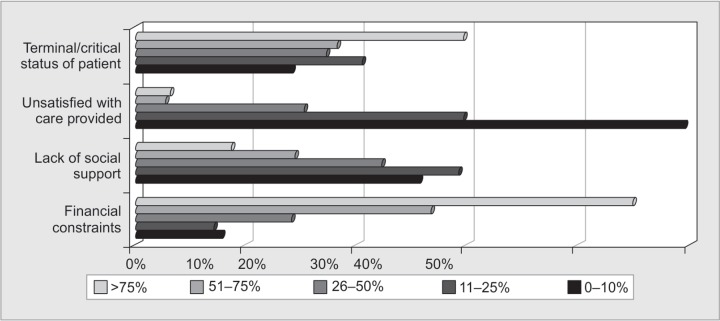
Distribution of probable reasons of LAMA patients

**Fig. 4:**
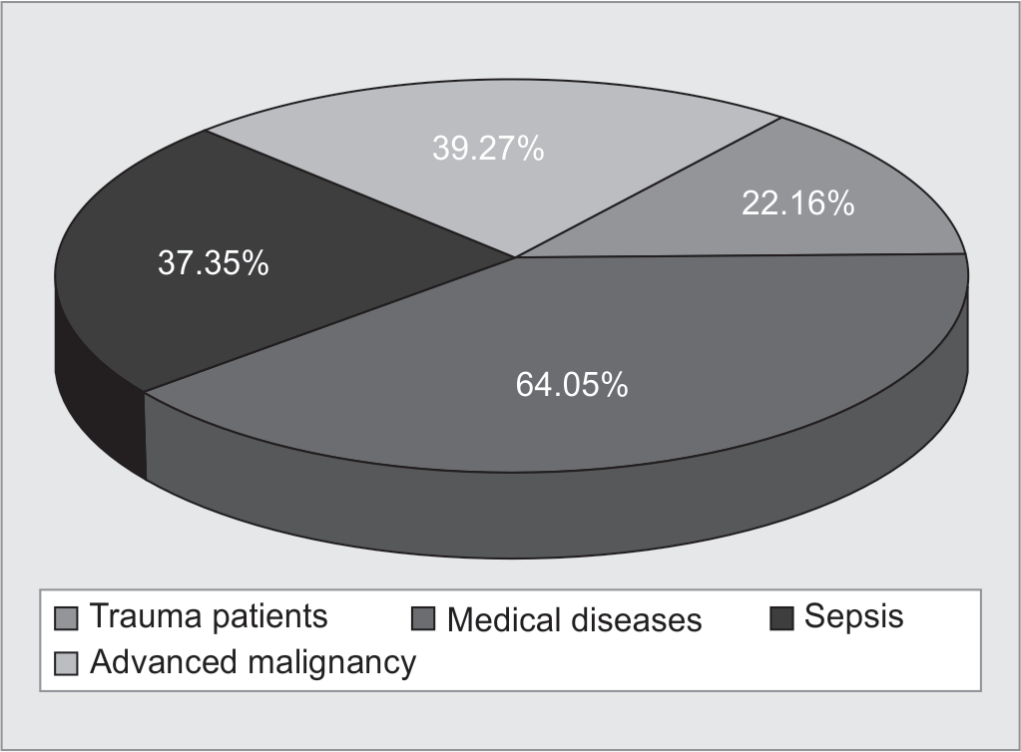
Distribution of LAMA patients according to diagnosis

There was variable response to the fourth section of reasons of LAMA. 80% of the respondents believed that financial constraints play a major role as one of the reasons of LAMA. According to the responses, the reasons for LAMA in decreasing order of frequency were financial constraints, critical or terminal condition of the patient, lack of social support and lastly, un-satisfaction with the medical care provided (Figure 3). In this study, 90% respondents believe that socioeconomic status of the patient has a role in deciding for LAMA especially in developing countries where as 58% disagree that iatrogenic complications play a role in LAMA. According to the responses, the most common diagnosis of patients going LAMA were medical disorders like cardiovascular, cerebro-vascular accidents or COPD, followed by advanced malignancy, sepsis and trauma in decreasing order of frequency (Figure 4).

On cross analysis there was no relation of LAMA with the type of institute, number of hospital/ICU beds, occupancy rate but was related significantly to the type of care provided by the hospital.

## DISCUSSION

Leaving against medical advice remains a major healthcare challenge that not only leads to series of negative health consequences but also elevated costs. There is evidence in literature that suggests increased rate of readmission among patients who leave hospital against medical advice as they fail to make full recovery during the first time they were treated^7^. Hence, they are associated with increased risk of morbidity and mortality^8^.

According to our survey, most clinicians believe that LAMA rate is least in children and higher in the elderly age group. But most studies in literature suggest that LAMA is a common phenomenon in the middle age group between 30-50 years^9^. These results are in accordance with retrospective and prospective trials in our own institute which show that 42% patients were in the 41-60 years age group. It may be due to difference in selection bias in the studies as most of these studies on LAMA have included patients with trauma or illicit drug use^10,11^. The influence of social and economical pressure on this group may also contribute to high rate of LAMA in this age group.

Our survey results show that 59.9% respondents think that patients leave the hospital within the first week. This is in coherence with a study in literature that document a mean duration of hospital stay of less than 3 days before leaving against medical advice^12^. The main reason to leave hospital in this study of trauma patients was to seek alternative medical advice or treatment therapy.

The results of our survey also suggest that very few patients who leave AMA have health insurance. In most low and medium income countries as ours social security services are non-existent, health policy of the state is not adequate and rate of unemployment is very high. In such conditions, families and care givers have to sustain the endless rising cost of medical care from their own pocket. Clinical experience of most of the respondents suggests that financial burden is an important reason to leave against medical advice especially in critically ill patients or those with terminal illness. Naderi et al suggested that 55 (3.84%) patients from emergency left AMA, of which 46 (84%) reported leaving because of financial restrictions. Thirty-nine (71%) respondents indicated that the medical bill was more that 25% of their annual income^13^.

The analysis of factors associated with LAMA including hospital characteristics and reasons of LAMA revealed that LAMA was associated only with type of care provided. The negative association between teaching status of a hospital and lower risk of self discharge has been shown in literature also^14^. The lower incidence of LAMA in tertiary care hospital providing highly specialized care or teaching hospitals is perhaps because patients fear that they might not be able to get better care elsewhere. Similarly, our survey also suggests that LAMA rates were less where high end intensive care is provided.

Some remedial methods suggested to decrease the incidence of self discharge include firstly a good national health policy by the state; secondly awareness among the people about advance directives; third, palliative and end of life care support practised uniformly among all hospitals and lastly improved communication between the patient, caregivers and the heathcare team. Adoption of national health insurance plan policy by the state does not cover all the costs but it clearly decreases the burden and makes healthcare including critical care more feasible for more people. This has been observed by the implication of Kenya's national health insurance plan^15^.

Some studies have attributed LAMA as a result of disagreements with planned treatments and dissatisfaction of the patients and caregivers with hospital facilities^16^. Health workers often face some difficulties in patient care attributable to limited facilities, being understaffed, busy schedules and uncooperative attitude of uneducated caregivers. This may lead to the disagreement and dissatisfaction. The key to success here is improved communication between patients and health team. Improved communication will clarify or reduce misconception and adverse attitude that sometimes lead to refusal of therapy.

Relatives constitute the primary caregivers and are involved in health decision-making at the expense of patients' consent. Literature reveals relatives were signatories up to 77.3% cases of LAMA but in reality all adults with the capacity to give consent “have the right of self determination and autonomy”^17,18^. Advance care planning includes the process of discussing, planning and communicating ones health care treatment and goals within the framework of a person's values. When they are codified into a legal document, it is termed as advanced directives (AD) or living will. This directive is valid and enforceable in most western countries but healthcare legislation in India has legally approved it in March, 2018 only^19^.

Lastly, providing specialized medical care to patients with serious, critical or terminal illness who have little or no hope of cure and recovery protects the dignity of a terminally ill dying patient. Physicians in many developing countries including ours have innovated LAMA for the sick, unconscious patients who are tortured to die without appropriate palliative care. The armamentarium of clinicians dealing with such patients includes the knowledge and competence on the aspects of withholding or withdrawing interventions and end of life support so as to alleviate the suffering of the dying patient and reduce incidence of LAMA. The practice of end of life care protocols makes dying without distressing symptoms and does not contribute to hasten death^20^.

Limitations of our study include that the response rate was low with only 22% respondents answering all the questions. Most had answered 60% of the questionnaire. A large proportion of the response (60%) was from the private set up (private medical colleges, corporate and private hospitals) so they reflect the idea soft he community attending these hospitals. Hence, further studies are still needed to explore this complex phenomenon.
